# Supplementary material for: How Gut Microbiome Perturbation Caused by Antibiotic Pre-Treatments Affected the Conjugative Transfer of Antimicrobial Resistance Genes
Source: Microorganisms. 2024 Oct 25;12(11):2148. doi: 10.3390/microorganisms12112148 (PMC11596856; doi:10.3390/microorganisms12112148)
Supplement: Supplementary file 1 [file microorganisms-12-02148-s001.zip › microorganisms-3246165-supplementary.pdf]

Supplementary Table S1. PCR primers and conditions for targeted gene quantification

Primer	Sequence (5'→3')	Primer (nM)	Annealing (A) & extension (E)	Amplicon size (bp)	Target gene	Reference
BACT1369F<br>PROK1492R	CGGTGAATACGTTCYCGG<br>GGWTACCTTGTTACGACTT	300	A: 56 ºC, 30 sec;<br>E: 72 ºC, 30 sec	123	small-subunit rRNA gene, *rrnS*	Suzuki *et al.*, 2000
Malo2-F<br>Malo2-Ra	GTATTGTTGATTAATGAGATCCG<br>ATATTACGCACGGAAACACGTT	250	A: 55 ºC, 30 sec;<br>E: 72 ºC, 30 sec	373	*Salmonella* invasion protein gene, *invA*	Malorny et al., 2001
blaCMY-2F<br>blaCMY-2R	AGG GAA GCC CGT ACA CGT T<br>GCT GGA TTT CAC GCC ATA GG	300	A: 52 ºC, 30 sec;<br>E: 72 ºC, 30 sec	205	β-lactam resistance gene, bla_{CMY-2}	Boyer & Singer, 2012
blaTEM-1F<br>blaTEM-1R	CATTTTCGTGTCGCCCTTAT<br>GGCGAAAACTCTCAAGGAT	200	A: 58 ºC, 30 sec;<br>E: 72 ºC, 30 sec	167	β-lactam resistance gene, bla_{TEM-1}	Resende. et al. 2014
intI1-F2<br>intI1-R2	TCGTGCGTCGCCATCACA<br>GCTTGTTCTACGGCACGTTTGA	400	A & E: 62 ºC, 60 sec	67	integrase class 1, *intI*1	Gaze *et al.*, 2011
sul1-F<br>sul1-R	GACTGCAGGCTGGTGGTTAT<br>GAAGAACCGCACAATCTCGT	200	A & E: 64 ºC, 60 sec	105	sulfonamide resistance gene 1, *sul*1	Marti *et al.*, 2014
strA-F<br>strA-R	TCAATCCCGACTTCTTACCG<br>CACCATGGCAAACAACCATA	400	A & E: 62 ºC, 60 sec	126	aminoglycoside-3″-phosphotransferase gene, *strA*	Walsh *et al.*, 2011

ermF-F	TCGTTTTACGGGTCAGCACTT	300	A & E: 61 °C, 60	182	erythromycin resistance	Knapp *et*
ermF-R	CAACCAAAGCTGTGTCGTTT		sec		gene locus F, *ermF*	*al*., 2010
tetQ-F	AGAATCTGCTGTTTGCCAGTG	500	A & E: 63 °C, 60	169	tetQ	Aminov *et*
tetQ-R	CGGAGTGTCAATGATATTGCA		sec			*al*. 2001
cfxA-F	TGACTGGCCCTGAATAATCT	1000	A: 55 °C, 30 sec;	301		Eitel *et*
cfxA-R	ACAAAAGATAGCGCAAATCC		E: 72 °C, 30 sec			*al*., 2013

References

Aminov, R.I.; Garrigues-Jeanjean, N.; Mackie, R.I. 2001. Molecular ecology of tetracycline resistance: development and validation of primers for detection of tetracycline resistance genes encoding ribosomal protection proteins. *Appl. Environ. Microbiol.* **2001**, *67*, 22-32. https://doi.org/10.1128/AEM.67.1.22-32.2001.

Boyer, T.C.; Singer, R.S. Quantitative measurement of bla_{CMY-2} in a longitudinal observational study of dairy cattle treated with ceftiofur. *Foodborne Pathog. Dis*. **2012**, *9* (11), 1022-1027. https://doi.org/10.1089/fpd.2012.1198 .

Eitel, Z.; Sóki, J.; Urbán, E.; Nagy. E. The prevalence of antibiotic resistance genes in Bacteroides fragilis group strains isolated in different European countries. Anaerobe **2013,** *21*, 43-49. https://doi.org/10.1016/j.anaerobe.2013.03.001.

Gaze, W.H.; Zhang, L.; Abdouslam, N.A.; Hawkey, P.M.; Calvo-Bado, L.; Royle, J.; Brown, H.; Davis, S.; Kay, P.; Boxall, A.B.A.; *et al*. Impacts of anthropogenic activity on the ecology of class 1 integrons and integron-associated genes in the environment. *ISME J*. **2011**, *5*, 1253-1261. https://doi.org/10.1038/ismej.2011.15.

Knapp, C.W.; Zhang, W.; Sturm, B.S.M.; Graham, D.W. Differential fate of erythromycin and beta-lactam resistance genes from swine lagoon waste under different aquatic conditions. *Environ. Pollut.* **2010**, *158*, 1506-1512. https://doi.org/10.1016/j.envpol.2009.12.020.

Malorny, B.; Bunge, C.; Helmuth, R. Evaluation of *Salmonella* spp . specific primer-sets for the validation within the food PCR project. **2001**, https://mobil.bfr.bund.de/cm/343/evaluation_of_salmonella_spp._specific_primer_sets_for_the_validation.pdf, accessed on October 16, 2019.

Marti, R.; Tien, Y.C.; Murray, R.; Scott, A.; Sabourin, L.; Topp, E. Safely coupling livestock and crop production systems: how rapidly do antibiotic resistance genes dissipate in soil following a commercial application of swine or dairy manure? *Appl. Environ. Microbiol.* **2014**, *80*, 3258-3265. https://doi.org/10.1128/AEM.00231-14.

Resende, J.A.; Diniz, C.G.; Silva, V.L.; Otenio, M.H.; Bonnafous, A.; Arcuri, P.B.; Godon, J.-J. Dynamics of antibiotic resistance genes and presence of putative pathogens during ambient temperature anaerobic digestion. *J. Appl. Microbiol.* **2014**, *117* (6), 1689-1699. https://doi.org/10.1111/jam.12653.

Suzuki, M.T.; Taylor, L.T.; DeLong, E.F. Quantitative analysis of small-subunit rRNA genes in mixed microbial populations via 5 '-nuclease assays. Appl Environ Microbiol. **2000**, *66*, 4605-4614. https://doi.org/10.1128/aem.66.11.4605-4614.2000.

Walsh, F.; Ingenfeld, A.; Zampicolli, M.; Hilber-Bodmer, M.; Frey, J.E.; Duffy, B. Real-time PCR methods for quantitative monitoring of streptomycin and tetracycline resistance genes in agricultural ecosystems. *J. Microbiol. Methods* **2011**, *86*, 150-155. https://doi.org/10.1016/j.mimet.2011.04.011.

Supplementary Table S2. Isolates of transconjugant from mice subjected to antibiotic pre-treatments followed by *Salmonella* infection.

Pre-treatment	1 day-post infection	3 day-post infection
None	Not detected	Not detected
Ampicillin	*E. coli* (SAMN40034289)[1]	Not detected
	E. coli (SAMN40034290)	
	E. coli (SAMN40034291)	
	E. coli (SAMN40034292)	
Streptomycin	*E. coli* (SAMN40034294)	*E. coli* (SAMN40034299)
	E. coli (SAMN40034295)	*E. coli* (SAMN40034300)
	E. coli (SAMN40034296)	*E. coli* (SAMN40034301)
	E. coli (SAMN40034297)	*E. coli* (SAMN40034302)
	S. Tyhpimurium (SAMN40034283)	*E. coli* (SAMN40034303)
	S. Tyhpimurium (SAMN40034284)	
	S. Tyhpimurium (SAMN40034285)	
	S. Tyhpimurium (SAMN40034286)	
	S. Tyhpimurium (SAMN40034287)	
	S. Tyhpimurium (SAMN40034288)	
Sulfamethazine	Not detected	Not detected

[1]BioSample accession number of isolates.

Supplementary Table S3. Weight changes and symptoms of mice subjected to antibiotic pre-treatments followed by *Salmonella* infection.

Pre-treatment	Weight change (%) from -7 day-post infection (dpi)						Remarks
	-7 dpi	-1 dpi	7 dpi	10 dpi	14 dpi	21 dpi	
None	100.0	105.6	122.2	122.2	122.2	111.1	No symptoms
None	100.0	105.3	94.7	94.7	100.0	94.7	No symptoms
None	100.0	105.9	111.8	111.8	111.8	111.8	No symptoms
None	100.0	111.1	122.2	122.2	122.2	105.6	No symptoms
None	100.0	100.0	100.0	100.0	105.6	100.0	No symptoms
Ampicillin	100.0	105.6	94.4	100.0	105.6	100.0	No symptoms
Ampicillin	100.0	118.8	106.3	112.5	118.8	118.8	No symptoms
Ampicillin	100.0	118.8	112.5	112.5	112.5	100.0	No symptoms
Ampicillin	100.0	116.7	100.0	105.6	111.1	100.0	No symptoms
Ampicillin	100.0	111.8	117.6	117.6	105.9	105.9	No symptoms
Ampicillin	100.0	105.6	94.4	100.0	111.1	94.4	No symptoms
Streptomycin	100.0	100.0	94.4	**77.8**	NA[1]	NA	Euthanized on 10 dpi as weight loss > 20%, no other symptoms
Streptomycin	100.0	100.0	100.0	106.3	106.3	106.3	No symptoms
Streptomycin	100.0	100.0	**73.7**	NA	NA	NA	Euthanized on 7 dpi as weight loss > 20%, no other symptoms
Streptomycin	100.0	105.3	89.5	**73.7**	NA	NA	Euthanized on 10 dpi as weight loss > 20%, no other symptoms
Streptomycin	100.0	100.0	**77.8**	NA	NA	NA	Euthanized on 7 dpi as weight loss > 20%, no other symptoms
Sulfamethazine	100.0	100.0	88.2	105.9	117.6	100.0	No symptoms
Sulfamethazine	100.0	94.1	94.1	105.9	111.8	105.9	No symptoms
Sulfamethazine	100.0	106.3	106.3	112.5	112.5	112.5	No symptoms
Sulfamethazine	100.0	94.4	88.9	100.0	116.7	100.0	No symptoms
Sulfamethazine	100.0	94.1	NA	NA	NA	NA	Found dead on 1 dpi, likely due to inoculation trauma

[1] NA = not available

Supplemental figure legends

Figure S1. Enumeration of *Salmonella* Typhimurium and *S.* Heidelberg (mean ± SE) in fecal samples from mice following the pre-treatment with no antibiotic (None), ampicillin (Amp), streptomycin (Strep) or sulfamethazine (Sulf); n = 5, 6, 5 and 5 for None, Amp, Strep and Sulf, respectively. ** indicate that the *Salmonella* abundance was significantly (p <0.01) different from those of the other treatment groups within the same serovar on the same day post infection (DPI).

Figure S2. Survival curves of C57BL/6 mice that were inoculated with *Salmonella* Typhimurium and *Salmonella* Heidelberg following the pre-treatment with no antibiotic (blue, n = 5), ampicillin (red, n = 6), streptomycin (green, n = 5), or sulfamethazine (purple, n = 5). Mortality occurred when mice either died unexpectedly or were euthanized due to morbidity. The asterisk (*) indicates $p < 0.05$ when comparing streptomycin (green curve) to other pre-treatments (blue, red or purple curve).

Figure S3. Pearson correlation ($P < 0.0001$) between the quantity of *sul1* (A), *intI1* (B), and *strA* (C) genes and the number of *Salmonella* in individual mouse fecal samples from all mice in this study with best fit line (solid) and 95% confidence bands (dotted).

Figure S4. Microbial species richness as assessed by the Chao1 index on each day post infection (DPI) from various per-treatment groups: None = no antibiotic, Amp = ampicillin, Strep = streptomycin, Sulf = sulfamethazine.

Figure S5. Principal coordinate analysis (PCoA) on Bray-Curtis dissimilarity of mouse gut microbiota derived from individual mice on each day post infection (DPI) from various pre-treatment groups: None = no antibiotic, Amp = ampicillin, Strep = streptomycin, Sulf = sulfamethazine.

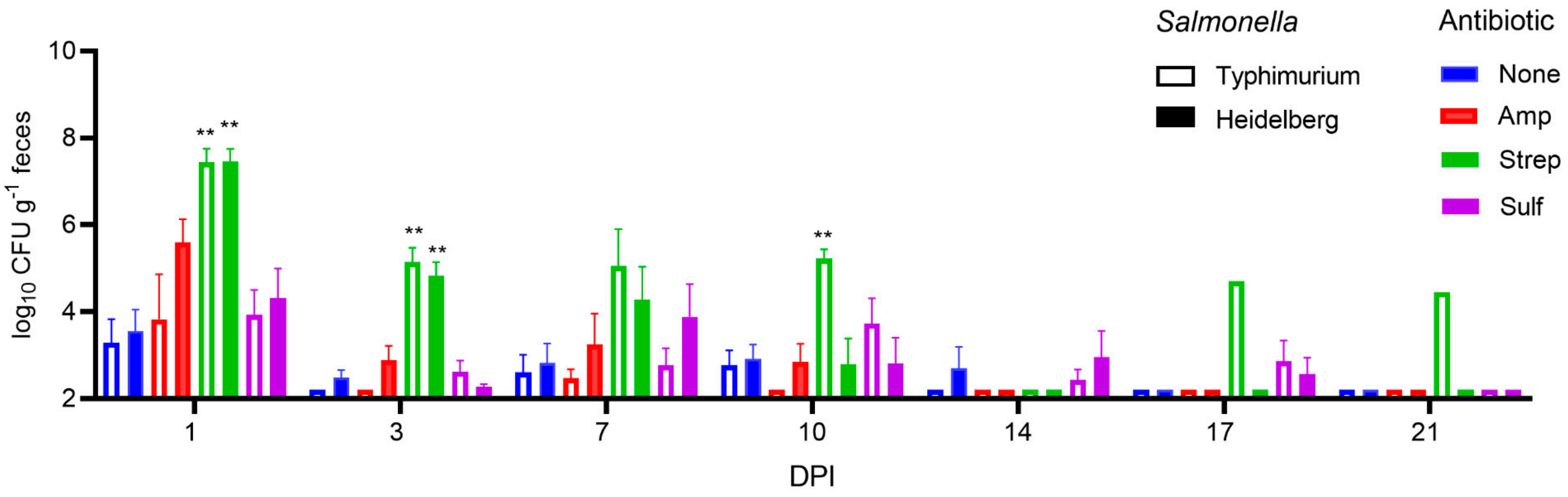

Salmonella
Typhimurium
Heidelberg
Antibiotic
None
Amp
Strep
Sulf
log10 CFU g-1 feces
10
8
6
4
2
**
**
**
**
**
1
3
7
10
14
17
21
DPI

Figure S1

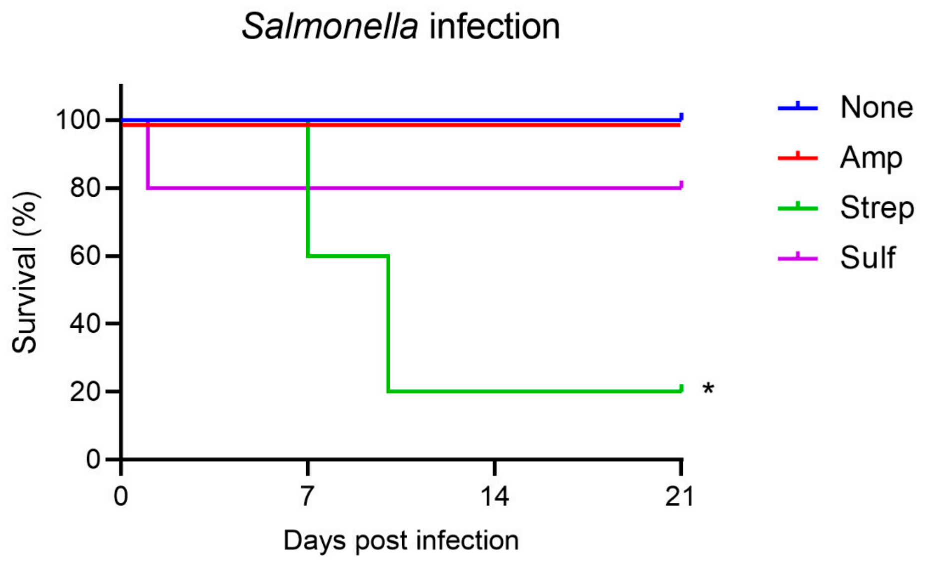
Salmonella infection
Survival (%)
100
80
60
40
20
0
0
7
14
21
Days post infection
*
None
Amp
Strep
Sulf

Figure S2

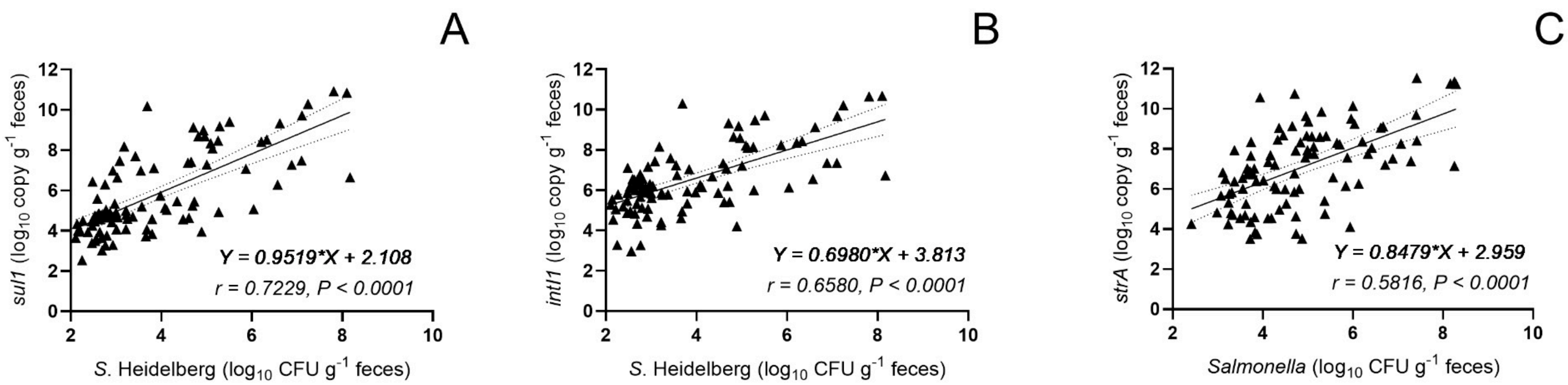
A
B
C
Y = 0.9519*X + 2.108
r = 0.7229, P < 0.0001
Y = 0.6980*X + 3.813
r = 0.6580, P < 0.0001
Y = 0.8479*X + 2.959
r = 0.5816, P < 0.0001
sul1 (log10 copy g-1 feces)
intI1 (log10 copy g-1 feces)
strA (log10 copy g-1 feces)
S. Heidelberg (log10 CFU g-1 feces)
Salmonella (log10 CFU g-1 feces)

Figure S3

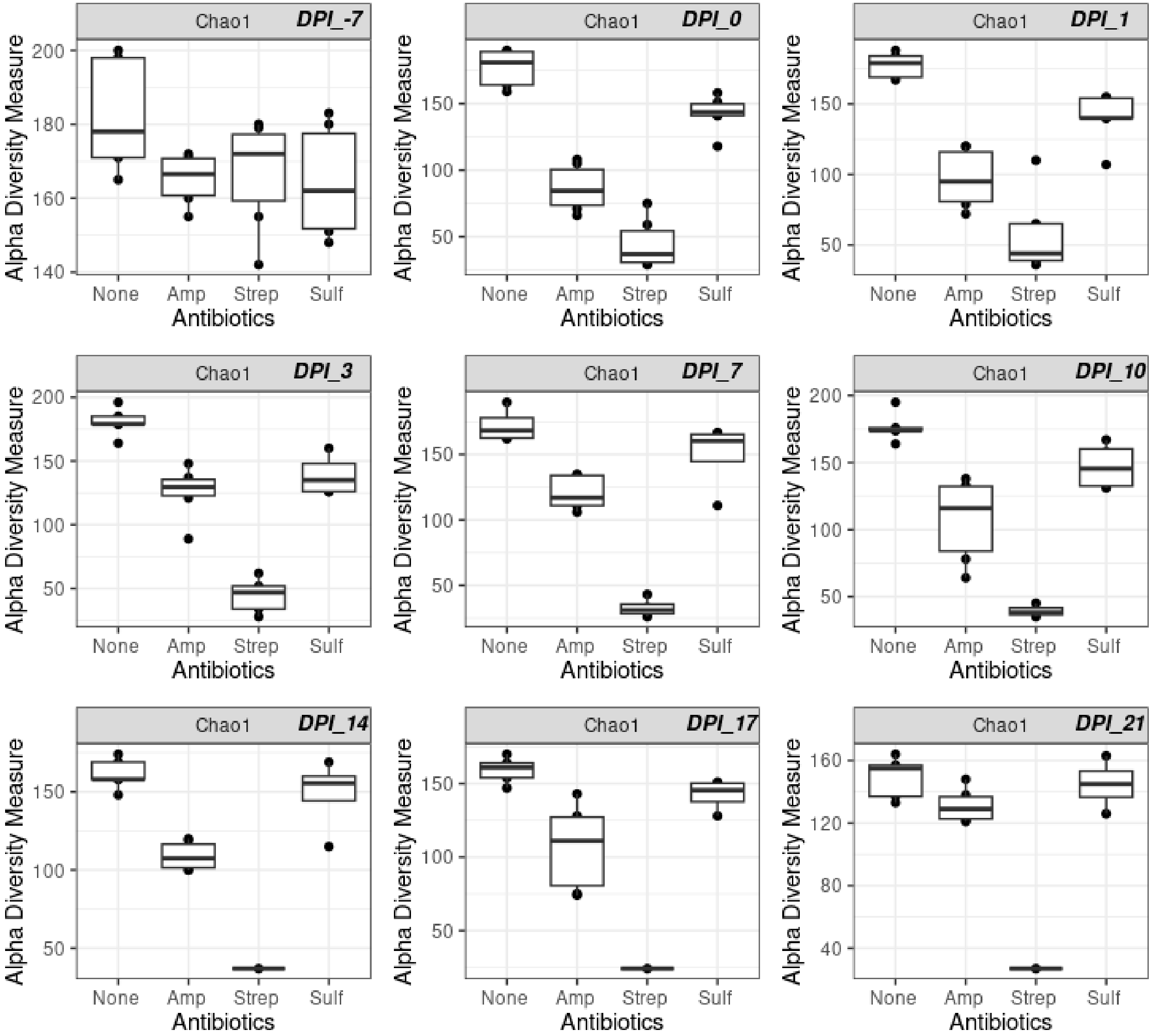
Chao1 DPI_-7
Chao1 DPI_0
Chao1 DPI_1
Chao1 DPI_3
Chao1 DPI_7
Chao1 DPI_10
Chao1 DPI_14
Chao1 DPI_17
Chao1 DPI_21
Alpha Diversity Measure
Antibiotics
None
Amp
Strep
Sulf

Figure S4

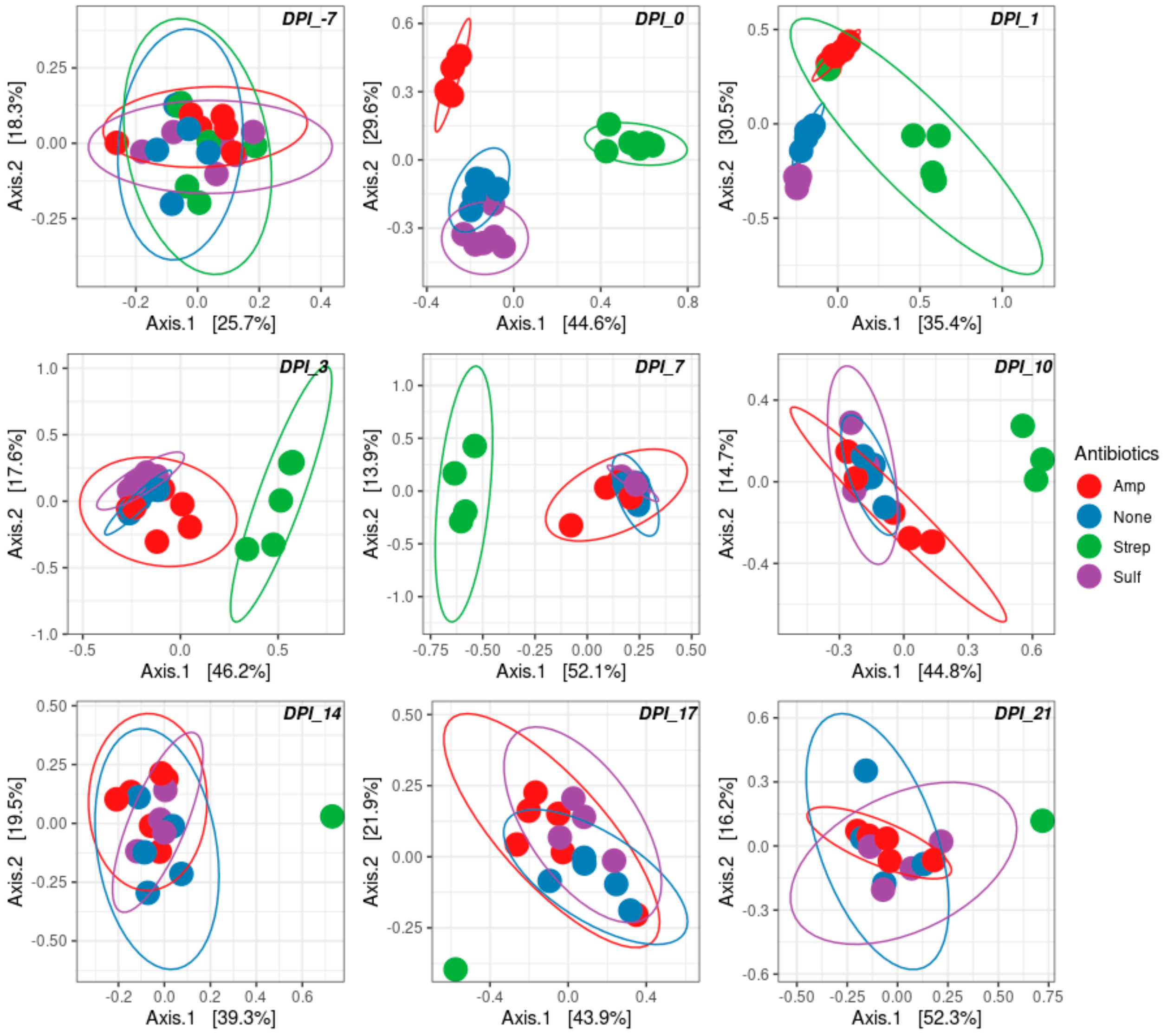
DPI_-7
DPI_0
DPI_1
DPI_3
DPI_7
DPI_10
DPI_14
DPI_17
DPI_21
Axis.1 [25.7%]
Axis.2 [18.3%]
Axis.1 [44.6%]
Axis.2 [29.6%]
Axis.1 [35.4%]
Axis.2 [30.5%]
Axis.1 [46.2%]
Axis.2 [17.6%]
Axis.1 [52.1%]
Axis.2 [13.9%]
Axis.1 [44.8%]
Axis.2 [14.7%]
Axis.1 [39.3%]
Axis.2 [19.5%]
Axis.1 [43.9%]
Axis.2 [21.9%]
Axis.1 [52.3%]
Axis.2 [16.2%]
Antibiotics
Amp
None
Strep
Sulf

Figure S5
